# PD-L1 and Survival in Solid Tumors: A Meta-Analysis

**DOI:** 10.1371/journal.pone.0131403

**Published:** 2015-06-26

**Authors:** Pin Wu, Dang Wu, Lijun Li, Ying Chai, Jian Huang

**Affiliations:** 1 Department of Thoracic Surgery, Second Affiliated Hospital, Zhejiang University School of Medicine, Zhejiang University, Hangzhou, 310009, China; 2 Department of Surgical Oncology, Second Affiliated Hospital, Zhejiang University School of Medicine, Zhejiang University, Hangzhou, 310009, China; 3 Cancer Institute (Key Laboratory of Cancer Prevention & Intervention, National Ministry of Education, Provincial Key Laboratory of Molecular Biology in Medical Sciences), Second Affiliated Hospital, Zhejiang University School of Medicine, Zhejiang University, Hangzhou, 310009, China; 4 Xixi Hospital of Hangzhou, Hangzhou, 310009, China; Vanderbilt University Medical Center, UNITED STATES

## Abstract

**Background:**

Numerous agents targeting PD-L1/PD-1 check-point are in clinical development. However, the correlation between PD-L1expression and prognosis of solid tumor is still in controversial. Here, we elicit a systematic review and meta-analysis to investigate the potential value of PD-L1 in the prognostic prediction in human solid tumors.

**Methods:**

Electronic databases were searched for studies evaluating the expression of PD-L1 and overall survival (OS) of patients with solid tumors. Odds ratios (ORs) from individual studies were calculated and pooled by using a random-effect model, and heterogeneity and publication bias analyses were also performed.

**Results:**

A total of 3107 patients with solid tumor from 28 published studies were included in the meta-analysis. The median percentage of solid tumors with PD-L1 overexpression was 52.5%. PD-L1 overexpression was associated with worse OS at both 3 years (OR = 2.43, 95% confidence interval (CI) = 1.60 to 3.70, P < 0.0001) and 5 years (OR = 2.23, 95% CI = 1.40 to 3.55, P = 0.0008) of solid tumors. Among the tumor types, PD-L1 was associated with worse 3 year-OS of esophageal cancer, gastric cancer, hepatocellular carcinoma, and urothelial cancer, and 5 year-OS of esophageal cancer, gastric cancer and colorectal cancer.

**Conclusions:**

These results suggest that expression of PD-L1 is associated with worse survival in solid tumors. However, the correlations between PD-L1 and prognosis are variant among different tumor types. More studies are needed to investigate the clinical value of PD-L1 expression in prognostic prediction and treatment option.

## Introduction

Co‑stimulatory and co‑inhibitory receptors play a pivotal role in T cell biology, as they determine the functional outcome of T cell receptor (TCR) signaling and immune surveillance [1]. However, the co-inhibitory mechanisms which are termed check-points elicited from cancer immunoediting can also facilitate cancer cell to escape from immunosurveillance [2]. Despite the complexity of cancer immunoediting [3], growing evidences suggest that the co-inhibitory receptors, such as cytotoxic T lymphocyte-associated antigen-4 (CTLA-4) and programmed death 1 (PD-1), play a crucial role in cancer immunoediting, especially in the equilibrium and escape stages [4].

Human programmed death-ligand 1 (PD-L1 or B7-H1), as a dominant ligand, plays a central role in antigen-specific T cell response mediating PD-1-dependent immune suppression. The abnormal expression of these ligands has been linked with prognosis and treatment response of multiple malignancies. For instance, overexpression of PD-L1 has been observed in different solid tumors including melanoma [5, 6], colorectal cancer [7], lung cancer [8–11], pancreatic carcinoma [12] and hepatocellular carcinoma [13–15]. Recently, clinic trials demonstrate that various cancer patients can get survival benefit from immune check-point targeted treatment [16].

Despite the clinical development of anti-PD-L1 therapies, the prognostic value of PD-L1 overexpression across different solid tumors is still unclear. Recently, it is reported that melanoma patients with PD-L1-expressing cells at the invasive tumor margin and inside tumors are more sensitive to anti-PD-1 therapy [17]. Another two studies showed that across multiple cancer types, responses were observed in patients with tumors expressing high levels of PD-L1, especially when PD-L1 expressed on tumor-infiltrating immune cells [18, 19]. The evidence suggests along with development of PD-L1/PD-1 targeted therapy, some biomarkers are needed for guiding individualized anti-PD-1 therapy option. It would be desirable to explore whether PD-L1 overexpression is associated with worse outcome. Moreover, PD-L1 overexpression may serve as a potential biomarker for prognostic prediction and PD-L1/PD-1 targeted treatment option in solid tumors.

Here, we present a meta-analysis evaluating the prognostic value of PD-L1 overexpression in solid tumor. The purpose of this study was to estimate the correlation of PD-L1 overexpression with survival in solid tumors, thereby shed more light on the development of PD-L1/PD-1 immune check-point targeted therapy and prognostic prediction.

## Materials and Methods

This meta-analysis was carried out in accordance with preferred reporting items for systematic reviews and meta-analyses statement [20].

### Identification and selection of studies

Pubmed, Web of Science and EBSCO were searched for studies evaluating the expression of PD-L1 and survival in solid tumors from 2002 to November 2014. The search terms included “programmed death-ligand 1” or “PD-L1” or “B7-H1” or “CD274” and “neoplasms” and the results were limited to human studies of solid tumors. In addition we used the entry “programmed death-ligand 1” or “PD-L1” or “B7-H1” or “CD274” and the name of each specific solid tumor to recognize additional studies. We identified a total of 350, 346 and 249 entries, respectively. Eligibility criteria were the measurement of PD-L1 by immunohistochemistry (IHC), availability of survival data for at least 3 years, and publication in English. Studies evaluating gene expression of PD-L1 measured by polymerase chain reaction were excluded from the analyses. Citation lists of retrieved articles were manually screened to ensure sensitivity of the search strategy. Study selection was based on the association of PD-L1 and survival. Inter-reviewer agreement was assessed using Cohen’s kappa coefficient. Disagreement was resolved by consensus.

### Endpoints of interest

The primary endpoints were overall survival (OS) at 3 and 5 years. Tumors were classified by PD-L1 expression status using cut-offs as defined by individual studies.

### Data collection process

Two authors (Pin Wu and Dang Wu) independently extracted information using predefined data abstraction forms. The following details were extracted by 2 reviewers (Pin Wu and Dang Wu): tumor type, number of patients, antibody used for the evaluation, technique used to quantify PD-L1, and cut-off to determine PD-L1 positivity. Survival data were extracted from tables or Kaplan—Meier curves for both PD-L1 negative or low (control group) and PD-L1 positive or high expression (experimental group).

### Data synthesis

The relative frequency of survival at 3 and 5 years between the control and experimental groups was expressed as an odds ratio (OR) and its 95% confidence interval (CI). A number of sensitivity analyses were prespecified.

### Statistical analysis

Data were extracted from the primary publications and analysed using RevMan 5.3 analysis software (Cochrane Collaboration, Copenhagen, Denmark). Estimates of ORs were weighted and pooled using the Mantel—Haenszel random effect model. Statistical heterogeneity was assessed using the Cochran’s Q and I^2^ statistics. Differences between subgroups were assessed using methods as previous described by Deeks et al [21]. Sensitivity analyses were carried out for different analytical methods and cut-offs for defining PD-L1 expression. All statistical tests were two-sided, and statistical significance was defined as P less than 0.05. No correction was made for multiple statistical testing.

## Results and Discussion

### Search results

**Fig 1:**
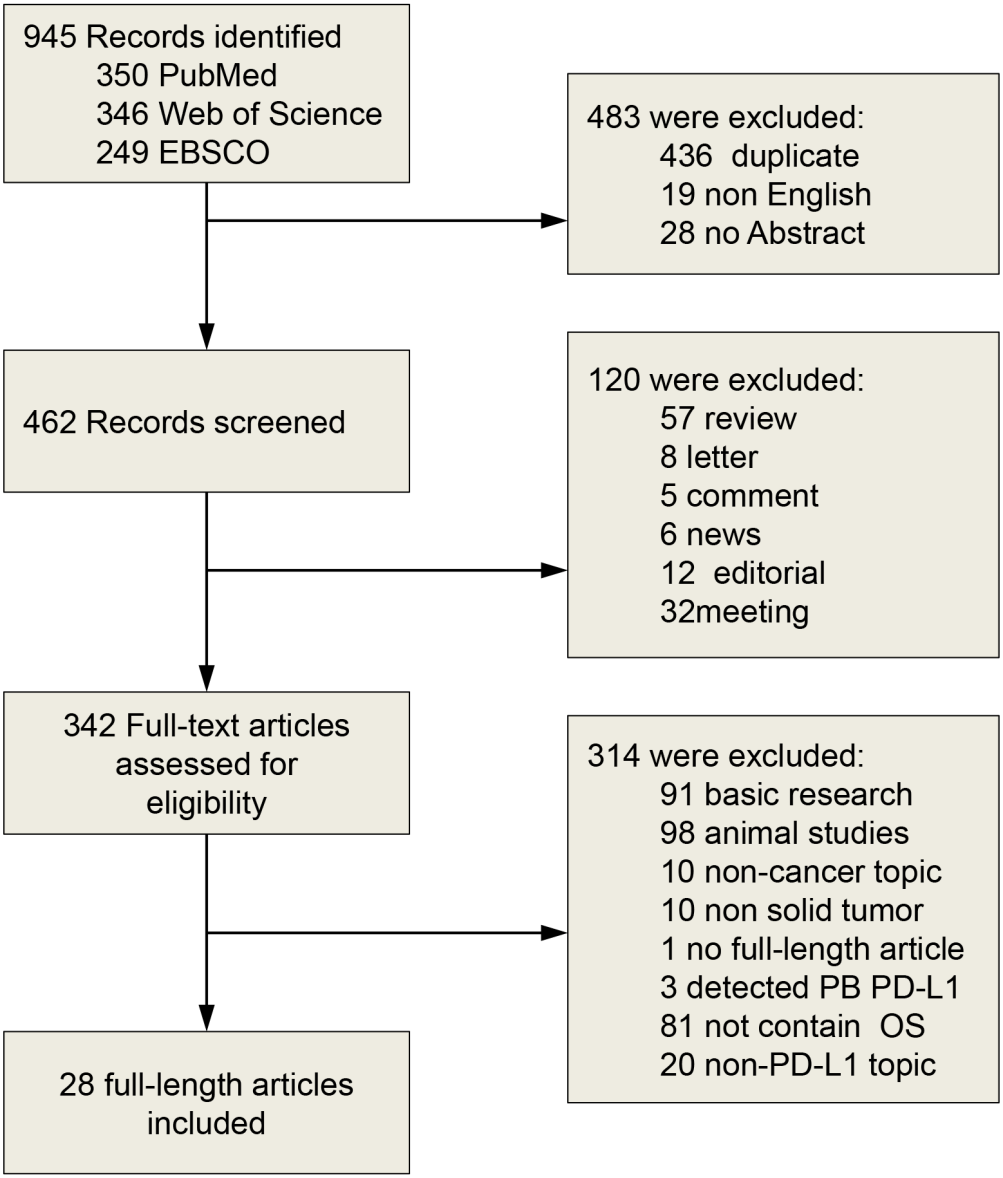
Flow diagram of literature search and study selection. PB, peripheral blood.

The search results have been shown in Fig 1. The primary literature research retrieved 945 records. After screening the title of citations, 603 records were excluded because of the non-relevance with the theme and duplicated literatures. Next, 314 citations were excluded after screening abstracts of the records. Then we carefully read the full text of the left citations and at last 28 studies were included.

### Description of studies

We identified 28 studies using IHC techniques for the assessment of PD-L1 expression. Characteristics of included studies are shown in Table 1. Four studies evaluated urothelial cancer, two evaluated colorectal cancer, three evaluated esophageal cancer, three evaluated gastric cancer, three evaluated hepatocellular carcinoma, four evaluated lung cancer, three evaluated melanoma, two evaluated oropharyngeal squamous cell carcinoma, and one each evaluated cervical cancer, glioblastoma, malignant mesotheliomas, ovarian cancer and pancreatic cancer. A total of 3107 patients were included in these studies.

**Table 1:** Characteristics of studies included in the meta-analysis.

Ref	Type of cancer	No.	Age, median (range)	Male/ Female	Stage	Follow up, months	PD-L1 (+/-) NO.	3-year OS (+/-)%	5-year OS (+/-)%
Boorjian, S. A et al.(2008)[22]	Urothelial cancer	167	NR	NR	I	> 120	27/140	53.2/82	41.2/68.6
Chen, L., et al. (2014)[23]	Esophageal cancer	99	59	76/23	I-IV	NR	82/17	40.4/63.6	22.2/63.6
Chen, X. L et al.(2009)[12]	Pancreatic cancer	40	54 (34–79)	26/14	I-IV	58.5	18/22	9.2/57.4	0/16.8
Chen, Y. B., et al. (2012)[24]	Lung cancer	120	NR	90/30	I-III	27	69/51	4.3/47.1	0/29.6
Cho, Y. A et al.(2011)[25]	Oral squamous cell carcinoma	45	NR	32/13	I-IV	NR	26/19	49.9/63.1	45.3/63.1
Gao, Q et al.(2009)[13]	Hepatocellular carcinoma	240	52 (18–81)	204/36	I-III	30	60/180	38.9/55	0/49
Geng, Y., et al. (2014)[26]	Gastric cancer	100	66.4 (30–87)	61/39	I-IV	>60	65/35	41.6/68.6	29.4/54.3
Hamanishi, J et al.(2007)[27]	Ovarian cancer	70	55 (26–78)	0/70	I-III	62.3	48/22	78.6/90	48.8/80
Hino, R et al.(2010)[5]	Melanoma	59	69.4 (25–87)	38/21	I-IV	NR	34/25	77.3/91.7	56.5/84.8
Hou, J. et al.(2014)[28]	Gastric cancer	111	NR(18–96)	75/36	I-IV	50	70/41	44.1/66.3	NR
Karim, R et al.(2009)[29]	Cervical carcinoma	115	48.5 (24–87)	NR	NR	60	22/93	82.1/84.2	82.1/75.4
Konishi, J et al.(2004)[8]	Lung cancer	52	66.4	35/17	I-IV	NR	26/26	NR	59/48
Liu, Y et al.(2013)[30]	Glioblastoma	17	NR (43–78)	11/6	NR	60	6/11	0/27	0/13.3
Loos, M et al.(2011)[31]	Esophageal cancer	101	64 (33–83)	NR	I-IV	75	37/64	52.7/83.5	32.8/69.5
Mansfield, A. S., et al. (2014)[32]	Malignant mesotheliomas	106	NR	90/16	NR	NR	42/64	2.1/10	0/4.8
Mu, C. Y et al.(2011)[9]	Lung cancer	109	NR	NR	I-III	NR	58/51	20.7/39.2	NR
Nakanishi, J et al(2007)[33]	Urothelial cancer	65	NR	47/18	I-IV	NR	46/19	68.8/100	68.8/100
Ohigashi, Y et al.(2005)[34]	Esophageal cancer	41	63 (46–73)	32/9	I-IV	25	18/23	18/53.8	18/45.3
Shi, S. J. et al.(2013)[7]	Colorectal cancer	143	NR	61/82	NR	60	64/79	51.2/70.6	43.5/58.8
Taube, J. M et al.(2012)[6]	Melanoma	99	53 (7–94)	NR	NR	100	43/56	84.2/62.5	84.2/62.5
Ukpo, O. C et al.(2013)[35]	Oral squamous cell carcinoma	181	55.8	158/23	I-IV	96	84/97	73.8/77.1	66.2/64.8
Velcheti, V et al.(2014)-Greek cohort [11]	Lung cancer	291	NR	NR	I-IV	NR	72/219	61.3/45.8	50.2/23.9
Velcheti, V et al.(2014)-Yale University cohort [11]	Lung cancer	153	NR	NR	I-IV	NR	55/98	71.5/45.3	53.3/36.8
Wang, Y et al.(2009)[36]	Urothelial cancer	50	61.7 (42–78)	40/10	NR	27.94	36/14	63.4/98.5	NR
Wu, C et al.(2006)[37]	Gastric cancer	102	55 (28–77)	75/27	NR	42	43/59	22.7/71	30.2/64.5
Wu, K et al.(2009)[15]	Hepatocellular carcinoma	71	48 (23–75)	65/6	I-IV	NR	35/36	58.3/83.3	40.5/68.8
Xylinas, E et al.(2014)[38]	Urothelial cancer	96	NR	NR	I-IV	NR	24/72	66.7/69.4	62.5/69.4
Yang, C. Y et al.(2014)[10]	Lung cancer	163	NR	54/109	I	71	65/98	100/100	96.8/99
Zhu, J., et al. (2014)[39]	Colorectal cancer	101	NR	53/48	NR	NR	55/46	NR	61.8/80.4

NR: Not reported.

### Evaluation and expression of PD-L1

A description of the antibodies used in the included studies is shown in Table 2. Various antibodies were used for the evaluation of PD-L1 expression. Seven studies used clone 5H1, five studies used clone MIH1, four studies used clone 2H11, two studies used clone 27A2 or ab82059, and one each used clone 236A/E7, NBP1-03220, ab58810 or primary antibody respectively. Four studies did not report the clone of PD-L1 antibody. The cut-off value for PD-L1 overexpression depended on the staining score and the method used. Among the group determined as PD-L1 overexpressed, the median overexpression of PD-L1 staining was 45.4%. Esophageal cancer, gastric cancer, and oropharyngeal squamous cell carcinoma had the highest expression of PD-L1, with more than 66.7% of tumors considered overexpression. Levels of PD-L1 overexpression in urothelial cancer, pancreatic carcinoma, colorectal cancer, melanoma, lung cancer, hepatocellular carcinoma, esophageal cancer, glioblastoma and cervical carcinoma ranged from 19% to 54.3%.

**Table 2:** Evaluation of human PD-L1 by immunohistochemistry (IHC) in the selected studies.

Ref	Type of cancer	PD-L1 + Tumor (%)	Antibody (Clone)	Cutoff for overexpression
Boorjian, S. A et al.(2008)[22]	Bladder cancer	12.40%	5H1	Positive: ≥5% tumor cells were positive for PD-L1 staining.
Chen, L., et al. (2014)[23]	Esophageal cancer	82.80%	NBP1-03220	IHC, presence of PD-L1 staining.
Chen, X. L et al.(2009)[12]	Pancreatic carcinoma	45.00%	2H11	Positive: ≥10% tumor cells were positive for PD-L1 staining.
Chen, Y. B., et al. (2012)[24]	Lung cancer	57.50%	236A/E7	IHC, IRS≥3 points, IRS = SI (staining intensity) × PP (percentage of positive cells). SI was determined as: 0, negative; 1, weak; 2, moderate; and 3, strong. PP was defined as: 0, negative; 1, 1–10% positive cells; 2, 11–50% positive cells; 3, 51–80% positive cells; and 4, more than 80% positive cells. IRS ≥ 3 points was regarded as PD-L1 positive expression.
Cho, Y. A et al.(2011)[25]	Oral squamous cell carcinoma	87.00%	ab82059	IHC, SID score; The proportion of stained cells in each field was assessed as: 0, no stained cells; 1, <25% stained cells; 2, 25–50% stained cells; and 3, >50% stained cells. Staining intensity was graded as: 0, negative staining; 1, light staining; 2, moderate staining; and 3, intense staining. A staining-intensity-distribution (SID) score was computed for each sample as follows: the score of the proportion of stained cells for each field was multiplied by the score of the staining intensity in that field to provide a SID score for the field. The mean of the five fields was the final SID score for the sample.
Gao, Q et al.(2009)[13]	Hepatocellular carcinoma	25.00%	MIH1	Positive: ≥75%; Three images of representative fields were captured under a Leica CCD camera DFC420 connected to a Leica DM IRE2 microscope (Leica Microsystems Imaging Solutions) at a magnification of 200 and saved as TIFF files using the Leica QWin Plus version 3 software. Images were analyzed with Image-Pro Plus version 6.2 software (Media Cybernetics) using a special function called measurement of integrated absorbance, which evaluate both the area and the intensity of the positive staining. With this function, integrated absorbance of all the positive staining of PD-Ls in each photograph was measured and its ratio to total area of each photograph was calculated as PD-Ls density. The average integrated absorbance value (integrated absorbance/total area) on each slide (three images) was used to represent a particular sample.
Geng, Y., et al. (2014)[26]	Gastric cancer	65.00%	2H11	IHC, IRS≥3, The intensity (I) of staining was graded on a scale of 0–3+, with 0 representing no detectable staining and 3+ representing the strongest staining. Four strongest staining regions were randomly selected under a 409 field. In each of the four regions, the rate of positive cell staining (R) under a 400 × field was calculated. R was defined as: 0, no staining; 1, ≤10% tumor cells with staining; 2, 11–50% tumor cells with staining; 3, 51–75% tumor cells with staining; and 4, >75% tumor cells with staining. Samples with scores<3 were considered as the negative and with scores ≥3 were considered as the positive. Histochemistry score = I × R.
Hamanishi, J et al.(2007)[27]	Ovarian cancer	68.60%	27A2	Positive: H-score ≥ 2; The expression of PD-L1 was evaluated according to the intensity of the staining and scored as follows: 0, negative; 1, very weak expression; 2, moderate expression; and 3, stronger expression. Cases with scores 0 and 1 were defined as the low-expression group, and cases with scores 2 and 3 were the high-expression group.
Hino, R et al.(2010)[5]	Melanoma	57.60%	27A2	RD value ≥90, Digital image analysis
Hou, J. et al.(2014)[28]	Gastric cancer	63.10%	ab82059	Positive: ≥10% tumor cells were postive for PD-L1 staining.
Karim, R et al.(2009)[29]	Cervical carcinoma	19.00%	5H1	IHC, presence of PD-L1 staining.
Konishi, J et al.(2004)[8]	Lung cancer	50.00%	MIH1	Positive: ≥11% tumor cells were postive for PD-L1 staining.
Liu, Y et al.(2013)[30]	Glioblastoma	35.29%	NR	IHC≥10 cells/field tumor cells were postive for PD-L1 staining
Loos, M et al.(2011)[31]	Esophageal cancer	73.30%	NR	IHC; H-score ≥ 4; Scores were given separately for the stained area and for the intensity of staining. Quantification was made as follows: 33% of the cancer cells or less, 1; more than 33% to 66% of the cancer cells, 2; and more than 66% of the cancer cells, 3. Intensity of staining was stated as absent or weak, 1; moderate, 2; and strong, 3. Each section had a final grade that derived from the multiplication of the area and intensity scores.
Mansfield, A. S., et al.(2014)[32]	Malignant mesotheliomas	40.00%	5H1	Positive: ≥5% tumor cells were postive for PD-L1 staining.
Mu, C. Y et al.(2011)[9]	Lung cancer	53.21%	Primary antibody	Positive: H-score ≥ median value; PD-L1 proteins were quantified using a visual grading system based on the extent of staining (by percentage of positive tumor cells graded on a scale of 0–3: 0 = none, 1 = 1–10%, 2 = 11–50%, 3 = 51–100%) and the intensity of staining (graded on a scale of 0–3: 0 = no staining, 1 = weak staining, 2 = moderate staining, 3 = strong staining). A semi-quantitative H-score was obtained by multiplying the grades of extent and intensity of staining. The median value of all the H-scores was chosen as the cutoff value for dividing the expression of proteins into high and low.
Nakanishi, J et al(2007)[33]	Bladder cancer	70.77%	MIH1	Positive: ≥12.2% tumor cells were postive for PD-L1 staining.
Ohigashi, Y et al.(2005)[34]	Esophageal cancer	43.90%	MIH1	Positive: ≥10% tumor cells were postive for PD-L1 staining.
Shi, S. J. et al.(2013)[7]	Colorectal cancer	44.80%	ab58810	IHC, presence of PD-L1 staining.
Taube, J. M et al.(2012)[6]	Melanoma	38.00%	5H1	Positive: ≥5% tumor cells were postive for PD-L1 staining.
Ukpo, O. C et al.(2013)[35]	Oral squamous cell carcinoma	46.40%	5H1	Positive: ≥5% tumor cells were postive for PD-L1 staining.
Velcheti, V et al.(2014)[11]	Lung cancer	86.00%	5H1	NR
Wang, Y et al.(2009)[36]	Bladder cancer	76.00%	NR	Positive: >10% tumor cells were postive for PD-L1 staining.
Wu, C et al.(2006)[37]	Gastric cancer	72.00%	2H11	IHC, presence of PD-L1 staining.
Wu, K et al.(2009)[15]	Hepatocellular carcinoma	42.20%	MIH1	NR
Xylinas, E et al.(2014)[38]	Bladder cancer	49.30%	5H1	NR
Yang, C. Y et al.(2014)[10]	Lung cancer	25.00%	NR	IHC ≥5% tumor cells were postive for PD-L1 staining
Zhu, J., et al. (2014)[39]	Colorectal cancer	39.90%	2H11	NR

NR: Not reported.

### Association of PD-L1 with survival

**Fig 2:**
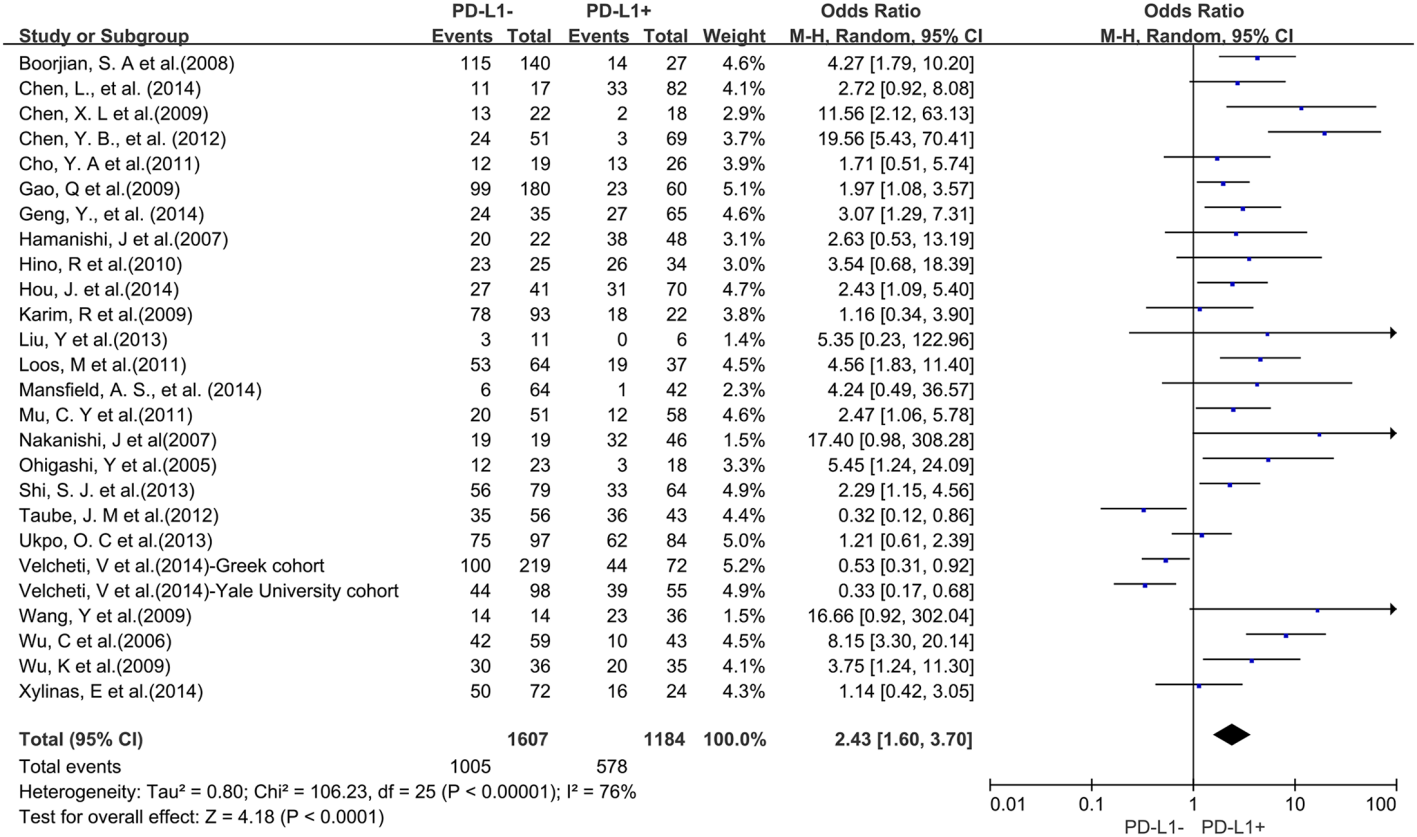
Forest plot describing the association between PD-L1 expression and 3-year OS of all patients with solid tumors.

A total of 25 studies reported data for OS at 3-years. Results showed that PD-L1 overexpression was associated with worse 3-year OS of solid tumors (OR = 2.43, 95% CI = 1.60 to 3.70, P < 0.0001) (Fig 2). There was significant heterogeneity among studies (Cochran’s Q P < 0.00001, I^2^ = 76%), so we conducted subgroup meta-analysis to explore whether the heterogeneity is due to different cancer types.

**Fig 3:**
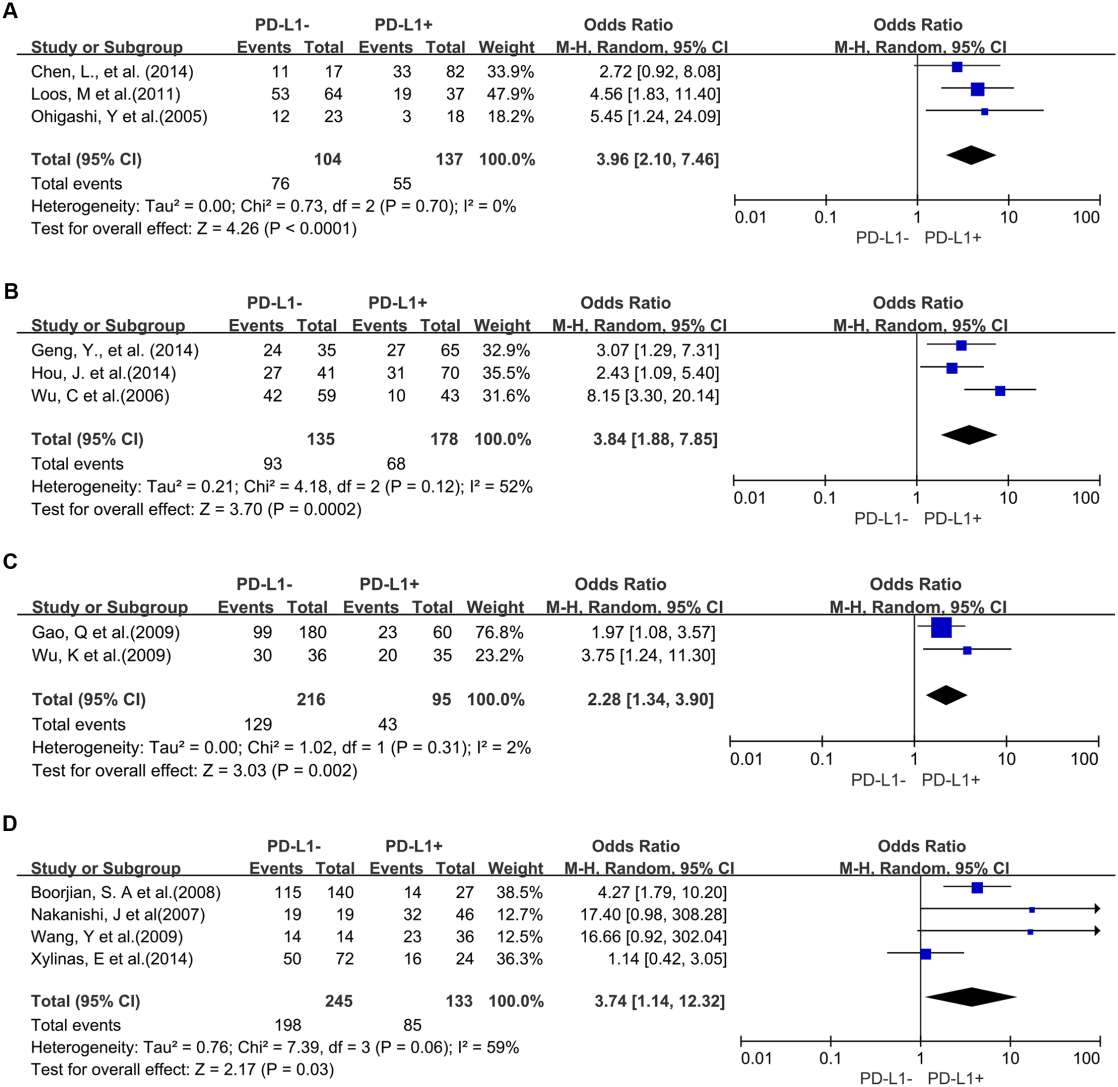
Forest plot describing subgroup analysis of the association between PD-L1 expression and 3-year OS of patients with esophageal cancer (A), gastric cancer (B), hepatocellular carcinoma (C) and urothelial cancer (D).

Three studies provided 3-year OS for lung cancer, 2 studies for hepatocellular carcinoma, 2 studies for melanoma, 2 studies for oropharyngeal squamous cell carcinoma, 4 studies for urothelial cancer, 3 studies for esophageal cancer, and 3 studies for gastric cancer. In the stratified analysis by cancer types, PD-L1 overexpression was associated with worse 3-year OS of esophageal cancer (OR = 3.96, 95% CI = 2.10 to 7.46, P < 0.0001) (Fig 3A), gastric cancer (OR = 3.84, 95% CI = 1.88 to 7.85, P = 0.0002) (Fig 3B), hepatocellular carcinoma (OR = 2.28, 95% CI = 1.34 to 3.90, P = 0.002) (Fig 3C) and urothelial cancer (OR = 3.74, 95% CI = 1.14 to 12.32, P = 0.03) (Fig 3D). However, there was no association between PD-L1 overexpression and 3-year OS of lung cancer (OR = 1.57, 95% CI = 0.38 to 6.48, P = 0.54) (S1A Fig), melanoma (OR = 0.97, 95% CI = 0.09 to 10.14, P = 0.98) (S1B Fig) and oropharyngeal squamous cell carcinoma (OR = 1.32, 95% CI = 0.73 to 2.38, P = 0.36) (S1C Fig).

**Fig 4:**
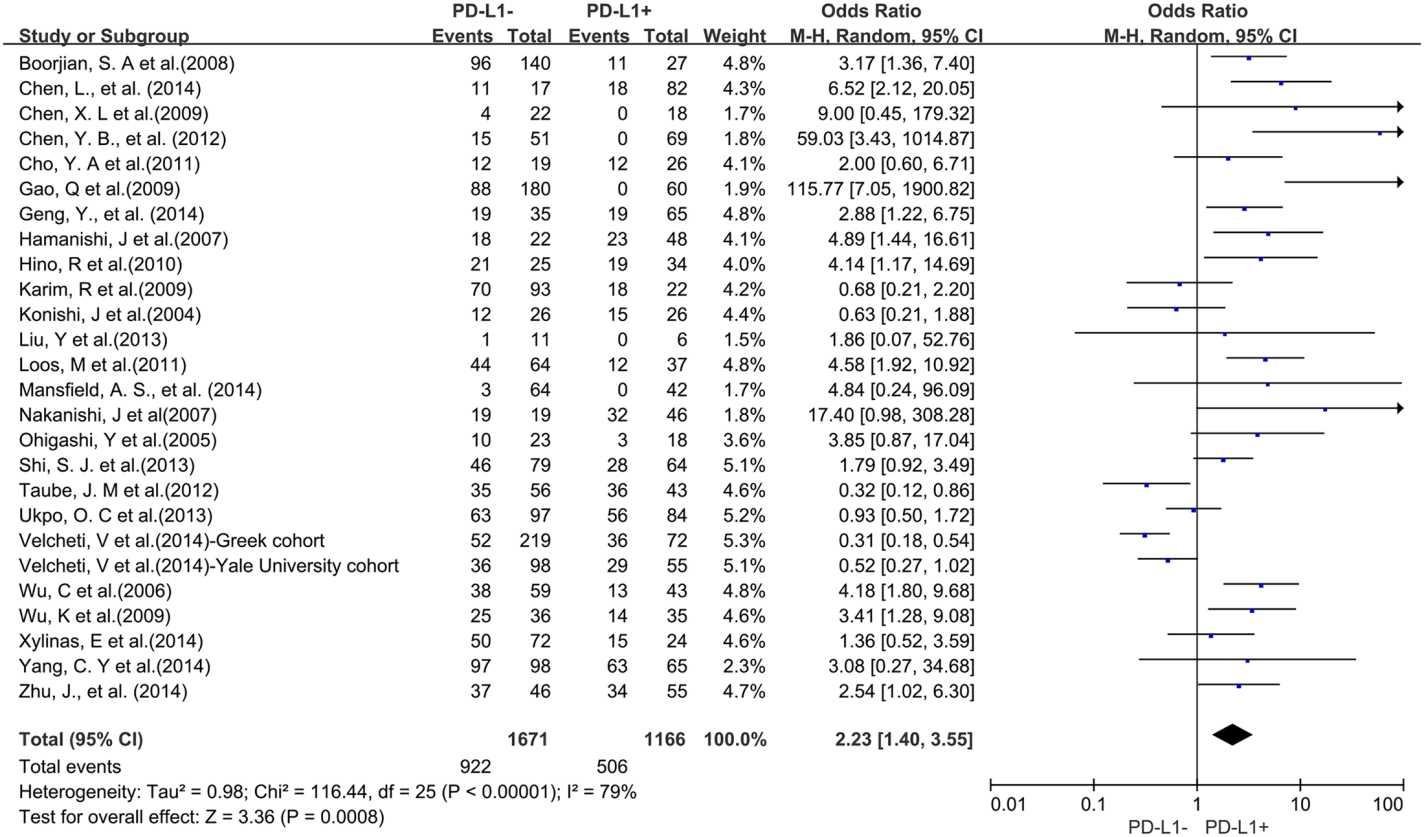
Forest plot describing the association between PD-L1 expression and 5-year OS of all patients with solid tumors.

A total of 25 studies reported data for OS at 5-years. Similar to the 3-year OS data, PD-L1 overexpression was significantly associated with worse 5-year OS of solid tumors (OR = 2.23, 95% CI = 1.40 to 3.55, P = 0.0008) (Fig 4). There was also high heterogeneity among studies (Cochran’s Q P < 0.00001, I^2^ = 79%), so we conducted subgroup meta-analysis according to different cancer types.

**Fig 5:**
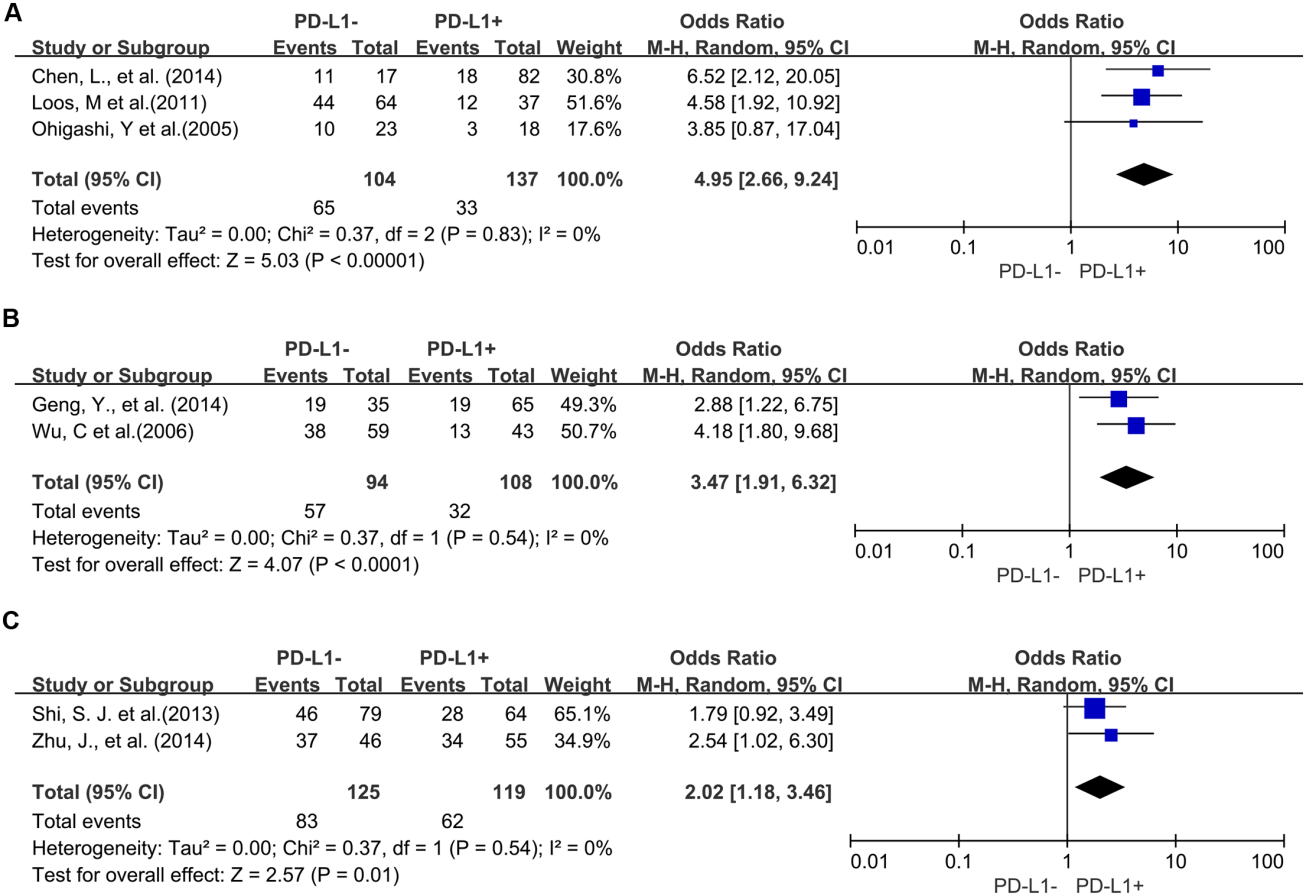
Forest plot describing subgroup analysis of the association between PD-L1 expression and 5-year OS of patients with esophageal cancer (A), gastric cancer (B) and colorectal cancer (C).

Four studies provided 5-year OS for lung cancer, 3 studies for hepatocellular carcinoma, 2 studies for melanoma, 2 studies for oropharyngeal squamous cell carcinoma, 3 studies for urothelial cancer, and 3 studies for esophageal cancer. PD-L1 overexpression was associated with worse 5-year OS of esophageal cancer (OR = 4.95, 95% CI = 2.66 to 9.24, P < 0.00001) (Fig 5A), gastric cancer (OR = 3.47, 95% CI = 1.91 to 6.32, P < 0.0001) (Fig 5B) and colorectal cancer (OR = 2.02, 95% CI = 1.18 to 3.46, P = 0.01) (Fig 5C). However, there was no significant association between PD-L1 overexpression and the 5-year OS of urothelial cancer (OR = 2.61, 95% CI = 1.00 to 6.85, P = 0.05) (S2A Fig), hepatocellular carcinoma (OR = 17.10, 95% CI = 0.19 to 1505.43, P = 0.21) (S2B Fig), lung cancer (OR = 0.91, 95% CI = 0.32 to 2.63, P = 0.60) (S2C Fig), melanoma (OR = 1.12, 95% CI = 0.09 to 13.64, P = 0.93) (S2D Fig) and oropharyngeal squamous cell carcinoma (OR = 1.13, 95% CI = 0.58 to 2.20, P = 0.71) (S2E Fig).

### Sensitivity analyses

Removal of studies which did not report the PD-L1 antibody clone did not influence results for 3-year or 5-year OS (OR = 2.26, 95% CI = 1.43 to 3.57; OR = 2.14, 95% CI = 1.31 to 3.51, respectively). However, removal of the studies that did not report the cut-off value of PD-L1 overexpression increased the association between PD-L1 and worse 3-year and 5-year OS compared with PD-L1 low expression (OR = 2.95, 95% CI = 2.03 to 4.28; OR = 2.70, 95% CI = 1.67 to 4.37, respectively). Exclusion of these studies did not reduce heterogeneity for 5-year OS (Cochran’s Q P = 0.0002, I2 = 60%; Cochran’s Q P < 0.00001, I2 = 68%, respectively).

### Discussion

PD-L1 overexpression has been observed in a substantial number of solid tumors. Moreover, numerous studies have demonstrated that PD-L1 plays a key role in cancer immune escape [40, 41]. A decade ago some studies reported that blockade of PD-L1 could improve antitumor immunity [42–44]. Recently, several studies show that therapies targeting PD-L1/PD-1 display clinical responses in patients with multiple cancer types expressing high levels of PD-L1 [17–19, 45–48]. These leading studies highlight that PD-L1 may serve as a biomarker for prognostic prediction and PD-L1/PD-1 targeted treatment option in solid tumors. In this study, we meta-analyzed the published data about the expression of PD-L1 in solid tumors and their association with survival for studies that evaluated PD-L1 by IHC.

Results showed that overexpression of PD-L1 was associated with worse 3-year OS for all studies analyzed except for one study of melanoma and lung cancer, respectively [6, 11]. Studies reporting 5-year OS data demonstrated that overexpression of PD-L1 is associated with worse outcome except for one study of cervical carcinoma [29], melanoma [6], oropharyngeal squamous cell carcinoma [35] and two study of lung cancer [8, 11]. Among the tumor types evaluated, esophageal cancer was the tumor type most linked with worse 3-year and 5-year outcome for patients with high levels of PD-L1 [31, 34]. Overall, PD-L1 overexpression was reported to be associated with worse 5-year outcome of patients with digestive tract-derived tumors such as esophageal cancer [23, 31, 34], gastric cancer [26, 37] and colorectal cancer [7, 39]. In addition, one study on hepatocellular carcinoma showed that PD-L1 expression on macrophages in tumors was significantly higher than paired normal tissues and correlated with tumor stage [14]. These results suggest that PD-L1 has an important role in the immune escape and progress of tumors especially digestive tract-derived.

A recent study reported that epithelial-originated cancer patients with positive expression of PD-L1 on tumor tissues were associated with significantly poorer OS when compared to those with negative expression of PD-L1 [49]. Consistently, our study showed that overexpression of PD-L1 in the tumor tissues of most epithelial-originated cancer types was associated with worse 3-year and 5-year OS except for lung cancer, melanoma and oral squamous cell carcinoma. However, recent clinic trials showed that patients with melanoma and lung cancer could acquire clinical benefit from anti-PD-L1 treatment [47, 48]. In our meta-analysis, we noted that the results of studies using different clone of PD-L1 antibodies were conflicting in melanoma [5, 6] (S1A and S1B Fig) and lung cancer [10, 11] (S2C Fig). The difference between technologies used in studies may partly account for the contradictory results. Further studies are needed to confirm the impact of antibodies on the results of studies.

Besides, the PD-1 expression state of tumor infiltrating lymphocytes is another key point of the PD-1/PD-L1-mediated tumor immune escape. A recent study suggests that pre-existing CD8^+^ T cells distinctly located at the invasive tumor margin are associated with expression of the PD-1/PD-L1 immune inhibitory axis and may predict response to therapy [17]. Moreover, another study showed that across multiple cancer types, responses were observed in patients with tumors expressing high levels of PD-L1, especially when PD-L1 was expressed on tumor-infiltrating immune cells [19]. The evidence implys that further study is needed to clarify the different prognostic and therapeutic prediction value of both PD-1 and PD-L1 expression on various cell types in solid tumor tissue.

## Conclusions

Our analyses show that overexpression of PD-L1 in solid tumor tissues, as measured by IHC, is associated with worse prognosis in different tumor types, which suggests that the development of strategies against the PD-L1/PD-1 axis would be a promising therapeutic approach for solid tumors. Moreover, further studies are required to investigate the potential role of PD-L1 expression in solid tumors for prognostic prediction as well as PD-L1 targeted treatment decision.

## Supporting Information

S1 Fig Forest plot describing subgroup analysis of the association between PD-L1 expression and 3-year OS of patients with lung cancer (A), melanoma (B) and oropharyngeal squamous cell carcinoma (C). (TIF)

S2 Fig Forest plot describing subgroup analysis of the association between PD-L1 expression and 5-year OS of patients with urothelial cancer (A), hepatocellular carcinoma (B), lung cancer (C), melanoma (D) and oropharyngeal squamous cell carcinoma (E). (TIF)

S1 PRISMA Checklist (DOC)
