# Supplementary figures and images for: PD-L1 and Survival in Solid Tumors: A Meta-Analysis
Source: PLoS One. 2015 Jun 26;10(6):e0131403. doi: 10.1371/journal.pone.0131403 (PMC4483169; doi:10.1371/journal.pone.0131403)

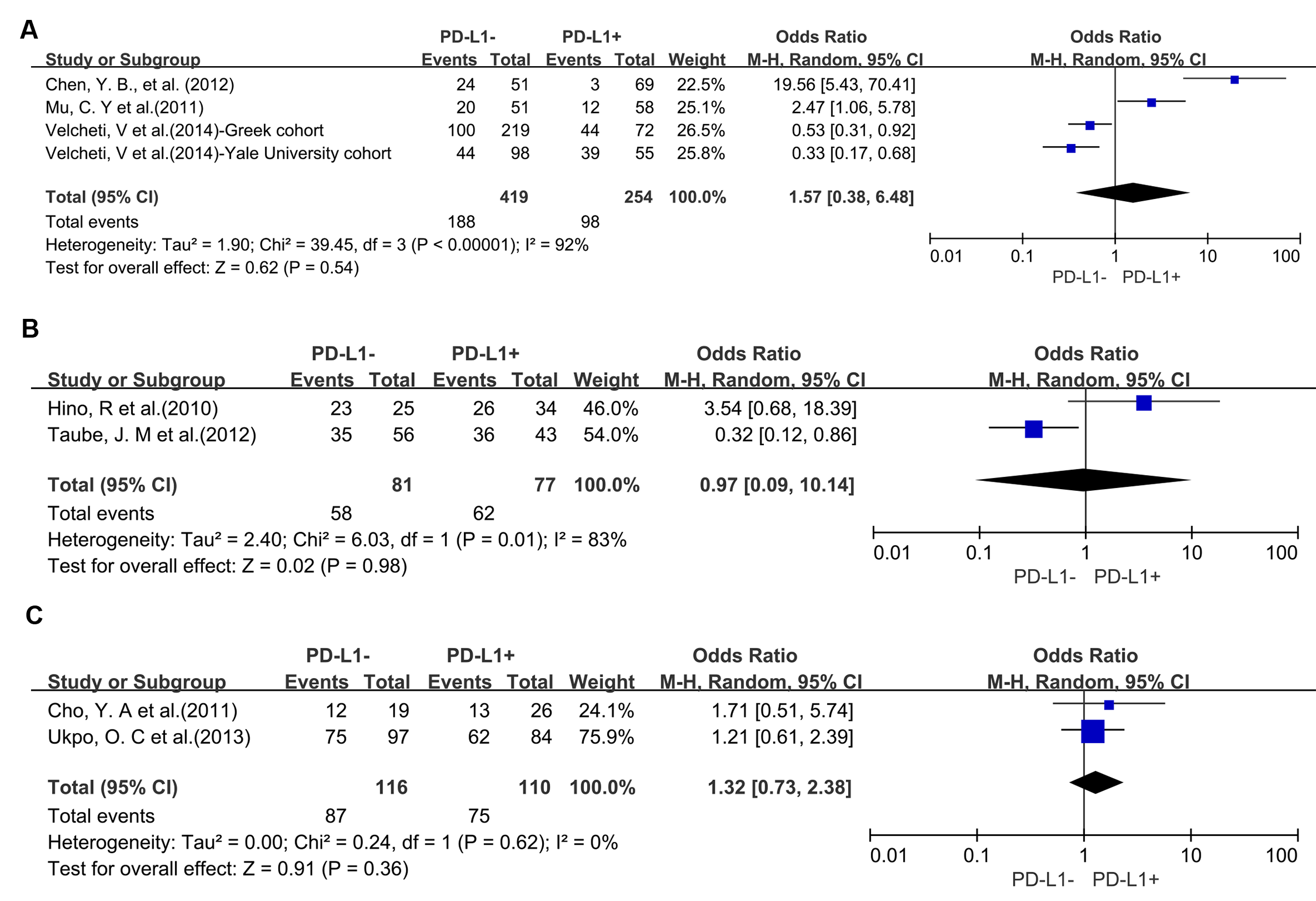

Supplement: S1 Fig — (TIF) [file pone.0131403.s001.tif]

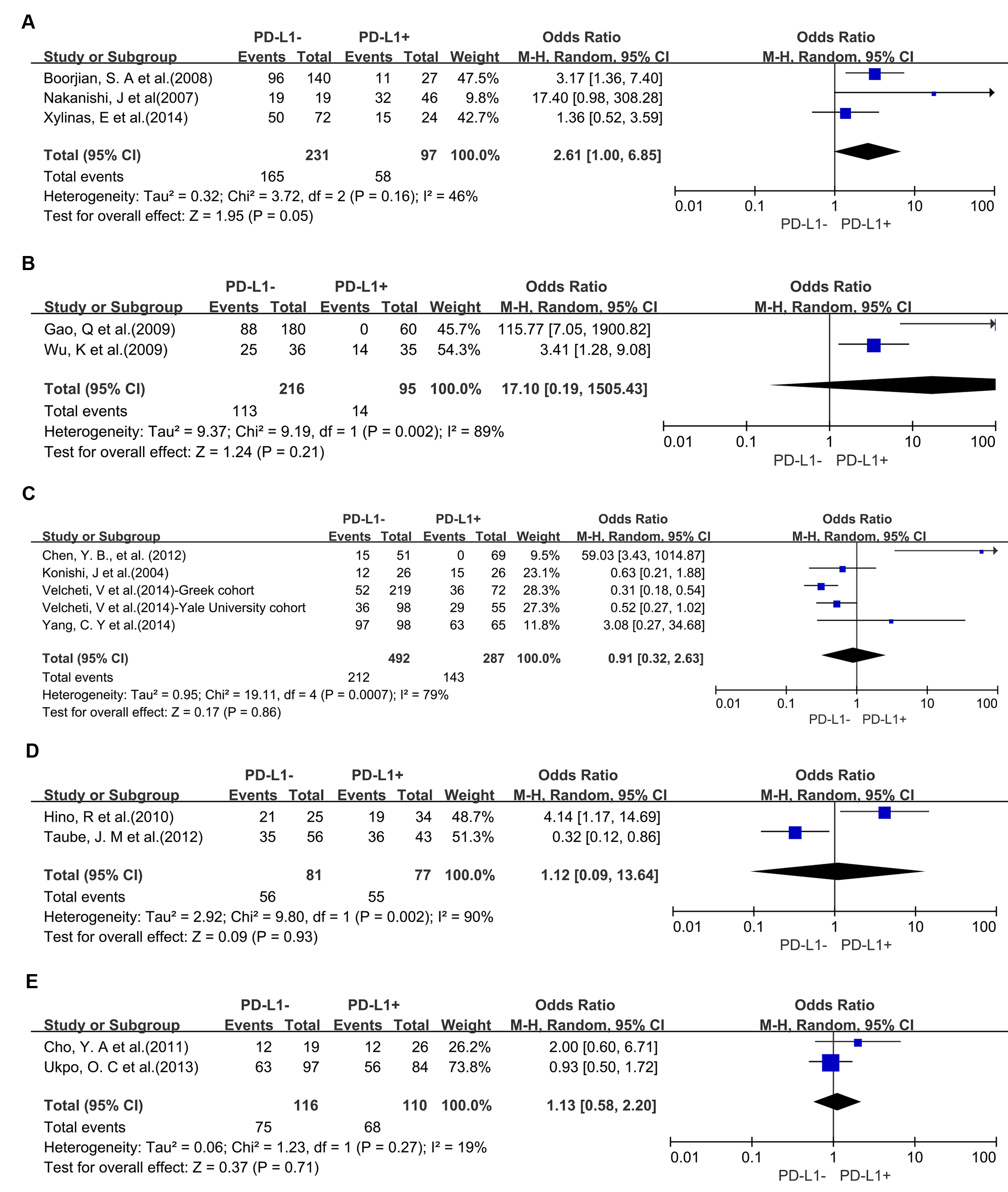

Supplement: S2 Fig — (TIF) [file pone.0131403.s002.tif]
